# A Durable Hierarchical ZnO/Epoxy Superhydrophobic Coating for Dual-Mode Drag Reduction of Surface and Underwater Vehicles

**DOI:** 10.3390/molecules31213896

**Published:** 2026-10-28

**Authors:** Ruoyu Sun, Yan Huang, Jing Zhao, Jiangpeng Qu

**Affiliations:** 1 School of Mechanical Engineering, Sichuan University of Science and Engineering, Yibin 644000, China; 2 College of Physical Education and Health Science, Yibin University, Yibin 644000, China; yan_huang@yibinu.edu.cn; 3 School of Mechanical Engineering, Southwest Jiaotong University, Chengdu 610031, China; jzhao@swjtu.cn (J.Z.);

**Keywords:** superhydrophobic coating, air-entrained cavity, air layer, drag reduction, durability

## Abstract

Superhydrophobic coatings can reduce hydrodynamic drag without external energy input, but their application in marine vehicles is hindered by limited durability and by studies addressing only a single navigation mode. Here, a durable ZnO/epoxy superhydrophobic coating with a hierarchical flower-like micro/nanostructure was fabricated by spray coating followed by chemical immersion, and its wettability, durability, and drag-reduction performance were systematically evaluated by model ship towing tests, steel-ball free-fall tests, interfacial friction measurements, and numerical simulations. The coating showed a water contact angle of 161.8 ± 3.1° and a roll-off angle below 1°, and remained superhydrophobic after 200 cm of sandpaper abrasion, 50 h of underwater immersion, 100 repeated water-entry cycles, and immersion in pH 1–11 solutions. The superhydrophobic model ship achieved drag-reduction efficiencies of up to 31%, while the 20 and 30 mm superhydrophobic steel balls achieved 19% and 28%, respectively. Interfacial friction measurements and numerical simulations revealed that the retained air layer induces wall slip on the water surface, whereas the entrained spindle-shaped cavity delays flow separation underwater, reducing both frictional and pressure drag. These results demonstrate a feasible coating strategy for energy-saving marine vehicles operating at or below the water surface.

## 1. Introduction

Surface and underwater vehicles are the primary platforms for ocean exploration, resource development, and maritime transport, and their energy consumption is dominated by hydrodynamic drag [1]. For underwater vehicles, the total drag consists of frictional drag, which arises from viscous shear within the boundary layer, and pressure (form) drag, which is caused by the adverse pressure gradient and flow separation around the hull [2,3]. For surface vehicles, viscous drag accounts for more than 80% of the total resistance, with the remainder being wave-making drag. Reducing these drag components is therefore essential for improving the speed, endurance, and energy efficiency of marine vehicles.

Over the past decades, various drag reduction techniques have been developed, including polymer additives [4,5], biomimetic riblets [6], compliant walls [7,8], microbubble injection [9,10], and Leidenfrost vapor layers [11]. The latter two rely on introducing a gas phase between the solid surface and water, thereby converting the solid–liquid contact into a solid–gas–liquid one. However, microbubble injection requires a continuous energy supply to generate and sustain the bubbles [10], while the Leidenfrost approach demands heating the surface beyond the boiling point of the surrounding liquid [11]; both contradict the original intention of energy saving. Superhydrophobic surfaces, which spontaneously trap an air layer (plastron) within their hierarchical micro/nanostructures upon immersion, offer a passive and energy-free alternative for establishing such a three-phase contact [12,13].

Whether superhydrophobic coatings can indeed reduce the drag of moving vehicles has long been debated. Reduced friction and large slip lengths have been demonstrated for flows over static superhydrophobic textures [14,15], whereas Steinberger et al. reported that trapped bubbles can act as an anti-lubricant and increase friction [16], and Su et al. [17] observed a higher underwater drag for superhydrophobic glass spheres than for hydrophilic ones. Cheng et al. [18] reconciled part of this controversy by demonstrating that the water capture status (Cassie vs. Wenzel) of a coating, which is accessible through water adhesive force measurements, determines whether drag is reduced or increased. On this basis, drag reduction has been verified for macroscopic model ships on the water surface [19,20,21] and for submarine models underwater [22], as well as for free-falling spheres, in which thin plastron layers trigger an early drag crisis [23] and entrapped air cavities streamline the body and suppress pressure drag [24]. More recently, superhydrophobic coatings have been integrated with anti-icing functions [25,26], combined with microbubble injection [27] and biomimetic riblets [28], and evaluated in shear-thinning fluids [29], reflecting the continued drive toward practical drag-reducing coatings [30].

Despite this progress, two limitations remain. First, most superhydrophobic coatings are evaluated under a single navigation mode—either on or under the water surface—although the governing drag mechanisms differ fundamentally: wall slip dominates the frictional drag of surface vessels, whereas cavity-mediated flow reorganization governs the pressure drag of underwater bodies. Whether one and the same coating can deliver both modes of drag reduction has rarely been examined. Second, durability remains the critical bottleneck for practical deployment: the trapped air layer dissolves over time [23,27], and the fragile micro/nanostructures are readily damaged by mechanical wear [31].

In this work, we report a durable hierarchical ZnO/epoxy superhydrophobic coating fabricated through a facile, low-cost spraying route followed by stearic acid modification, and we systematically evaluate its drag reduction performance under both surface and underwater navigation conditions. The design of this coating follows the established principles for superhydrophobic coatings deposited from dispersions [32]. The particulate phase of the sprayed ZnO/epoxy dispersion provides the micro/nano-scale roughness, while the subsequent chemisorption of the low-surface-energy stearic acid secures a stable Cassie–Baxter state, which is the prerequisite for retaining the drag-reducing air layer. The coating retains its superhydrophobicity after 200 cm of sandpaper abrasion, 50 h of underwater immersion, 100 repeated water-entry cycles, and exposure to pH 1–13 solutions. For surface navigation, model-ship towing tests are combined with weak solid–liquid interfacial friction measurements and FLUENT simulations to quantify the slip-induced reduction of frictional drag. For underwater navigation, free-fall tests of coated steel spheres with different diameters, high-speed imaging of the entrapped air cavities, and STAR-CCM+ simulations are employed to elucidate the cavity-mediated reduction of pressure drag. The results establish a single robust coating that delivers dual-mode drag reduction and provide design guidelines for energy-efficient marine vehicles operating at or below the water surface.

## 2. Theory

### 2.1. Drag Coefficient and Drag-Reduction Efficiency of a Free-Falling Sphere

At terminal velocity, the submerged weight of the sphere is balanced by the hydrodynamic drag:

(1) $$(m_{s}-m_{w})g=\frac{\pi \rho_{w}C_{D}D^{2}v_{t}^{2}}{8}$$

where *m*_s_ and *m*_w_ are the masses of the steel ball and the displaced water, respectively. *D* is the ball diameter; *ρ*_w_ is the density of water; and *v*_t_ is the terminal velocity. Substituting the mass relations *m*_s_ = (π/6)*ρ*_s_*D*^3^ and *m*_w_ = (π/6)*ρ*_w_ *D*^3^ into Equation (1), the drag coefficient is obtained directly from the measured terminal velocity:

(2) $$C_{D}=\frac{4(\rho_{s}-\rho_{w})Dg}{3\rho_{w}v_{t}^{2}}$$

The particle Reynolds number is defined as:

(3) $$Re=\frac{\rho_{w}v_{t}D}{\mu }$$

and the underwater drag-reduction efficiency is calculated as:

(4) $$DR=\frac{C_{D,s}-C_{D,\mathrm{sh}}}{C_{D,s}}\times 100\%$$

where *C*_D,s_ and *C*_D,sh_ are the drag coefficients of the smooth and superhydrophobic balls, respectively.

### 2.2. Drag Coefficient and Drag-Reduction Efficiency of the Model Ship

The drag coefficient of the model ship was calculated as [11]:

(5) $$C_{D}=\frac{2F_{D}}{(\rho U_{T}^{2}A)}$$

where *F*_D_ is the total hydrodynamic drag, *v* is the ship velocity, and *A* is the characteristic (wetted) area. Since the towing force *F*_T_ is the only driving force, *F*_D_ equals *F*_T_ only when the ship moves at a constant (terminal) velocity; therefore, all drag coefficients in this work were evaluated at the terminal velocity. The surface drag-reduction efficiency was then determined by:

(6) $$DR=\frac{C_{D,s}-C_{D,\mathrm{sh}}}{C_{D,s}}\times 100\%$$

where *C*_D,s_ and *C*_D,sh_ are the drag coefficients of the smooth and superhydrophobic surfaces, respectively.

For the solid–liquid interfacial friction test, the frictional force was derived from the strain *ε* measured by the strain gauge. The normal stress *σ* at the gauge position of the cantilever beam is:

(7) σ=εE

where *E* is the elastic modulus of the beam. For a beam under bending, the stress–moment relation is:

(8) $$\sigma =\frac{My}{I_{z}}$$

where *M* is the bending moment, *y* is the distance from the neutral axis (*y* = *h*/2 at the beam surface), and *I*_z_ is the second moment of area about the z-axis. Although the beam is subjected to transverse loading rather than pure bending, its length-to-height ratio (*L*/*h*) is much larger than 5, so the pure-bending relations remain applicable. *I*_z_ and *M* are given by:

(9) $$I_{z}=\int y^{2}dA=\frac{{bh}^{3}}{12}$$

(10) $$M=\frac{\sigma I_{z}}{y}$$

where b and h are the width and height of the beam cross-section, *F* is the frictional force applied at the beam end, and *L* is the distance from the gauge to the loading point. Combining Equations (7)–(10), the frictional force is obtained as:

*F* = *Eεbh*^2^/(6*L*) (11)

and the corresponding drag-reduction efficiency of the superhydrophobic coating is:

*DR* = (*F*_1_ − *F*_2_)/*F*_1_ × 100% (12)

where *F*_1_ and *F*_2_ are the frictional forces measured on the smooth and superhydrophobic samples, respectively.

## 3. Results

### 3.1. Characterization of Superhydrophobic Coatings

**Figure 1:**
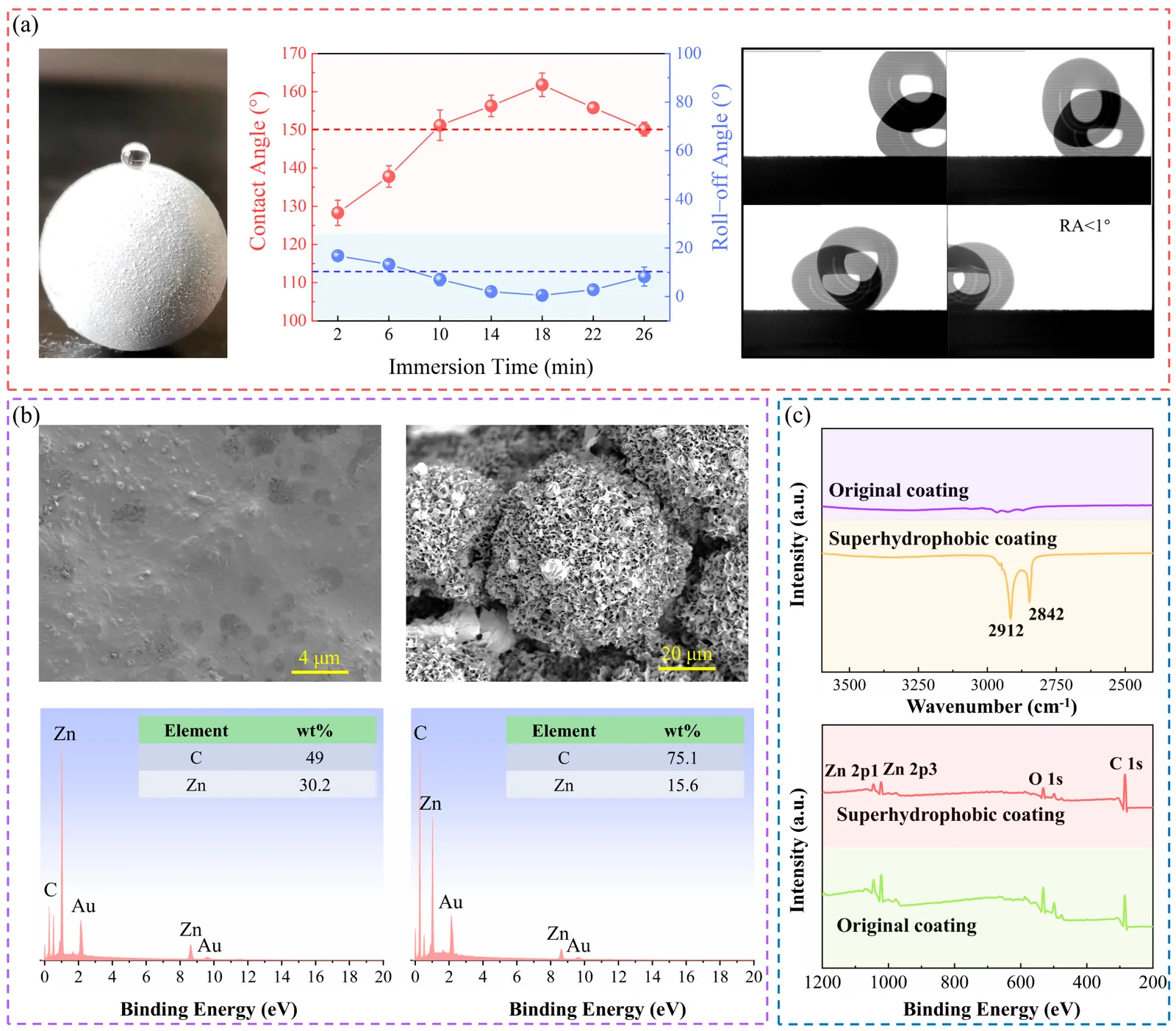
Characterization of the coatings. (**a**) CA of the coating as a function of immersion time in the acetic acid/stearic acid/ethanol solution (inset: a water droplet rolling off the coated steel ball without adhesion); (**b**) Field-emission scanning electron microscopy (SEM) images and EDS results of the original ZnO/epoxy coating and the superhydrophobic coating; (**c**) FTIR and XPS spectra of the original and superhydrophobic coatings.

The wettability of the coating was first optimized by varying the immersion time in the acetic acid/stearic acid/ethanol solution. Figure 1a shows the water contact angle (CA) of the coating as a function of immersion time (2–26 min). The CA increased rapidly with immersion time and reached a maximum of 161.8 ± 3.1° at 18 min, with a roll-off angle (RA) below 1°; further prolonging the immersion led to a slight decrease in CA, probably due to excessive etching of the microstructures by acetic acid. Accordingly, 18 min was adopted as the optimal immersion time for all subsequent samples. As shown in the inset of Figure 1a, water droplets readily rolled off the coated steel ball without any adhesion, confirming the excellent water repellency of the coating.

The superior superhydrophobicity originates from the hierarchical micro/nanostructures formed during the immersion process. Figure 1b compares the surface morphologies of the original (unmodified) and superhydrophobic coatings. The original ZnO/epoxy coating exhibited a relatively smooth surface, whereas the superhydrophobic coating was uniformly covered by microspheres with an average diameter of ca. 45 μm, each of which was assembled from porous nanosheets. The white-light interferometry result (Figure S1) further revealed an arithmetical mean height surface roughness (Sa) of 12.1 μm (Rq = 15.1 μm, Rt = 107.6 μm) over a scan area of 4.56 × 3.42 mm^2^, quantitatively confirming the micro-scale roughness introduced by the immersion process. High-magnification SEM (Figure S2) resolves the nanoscale features of the coating: the microspheres are assembled from an interconnected network of porous nanosheets with wall thicknesses of ca. 30–60 nm, i.e., more than two orders of magnitude below the micro-scale roughness (Sa = 12.1 μm) quantified by white-light interferometry. The two techniques are thus complementary: interferometry quantifies the micro-level of the hierarchy, while SEM resolves and quantifies the nano-level. Such dual-scale roughness is particularly important for the present fluorine-free system, whose modifier (stearic acid) has a higher surface energy than fluorinated alternatives, because it amplifies the intrinsic hydrophobicity into superhydrophobicity by stabilizing the Cassie–Baxter state [32].

The surface chemical modification was verified by energy-dispersive X-ray spectrometer (EDS), Fourier-transform infrared spectroscopy (FTIR) and X-ray photoelectron spectroscopy (XPS) analyses. The energy dispersive spectroscopy (EDS) results revealed that the carbon content increased from 49.0 wt% in the original coating to 75.1 wt% in the superhydrophobic coating, while the zinc content decreased from 30.2 wt% to 15.6 wt%, indicating the grafting of carbon-rich species onto the coating surface. In the FTIR spectra (Figure 1c), two new characteristic peaks appeared for the superhydrophobic coating, which are assigned to the asymmetric (2915 cm^−1^) and symmetric stretching vibrations of the C–H bonds in the long alkyl chains of stearic acid (2847 cm^−1^). The XPS results further confirmed the change in surface chemical bonds after modification. Together, these results demonstrate that stearic acid was successfully grafted onto the hierarchically structured coating, which lowers the surface energy and, in combination with the micro/nano roughness, accounts for the observed superhydrophobicity [32].

### 3.2. Durability and Stability of the Superhydrophobic Coating

**Figure 2:**
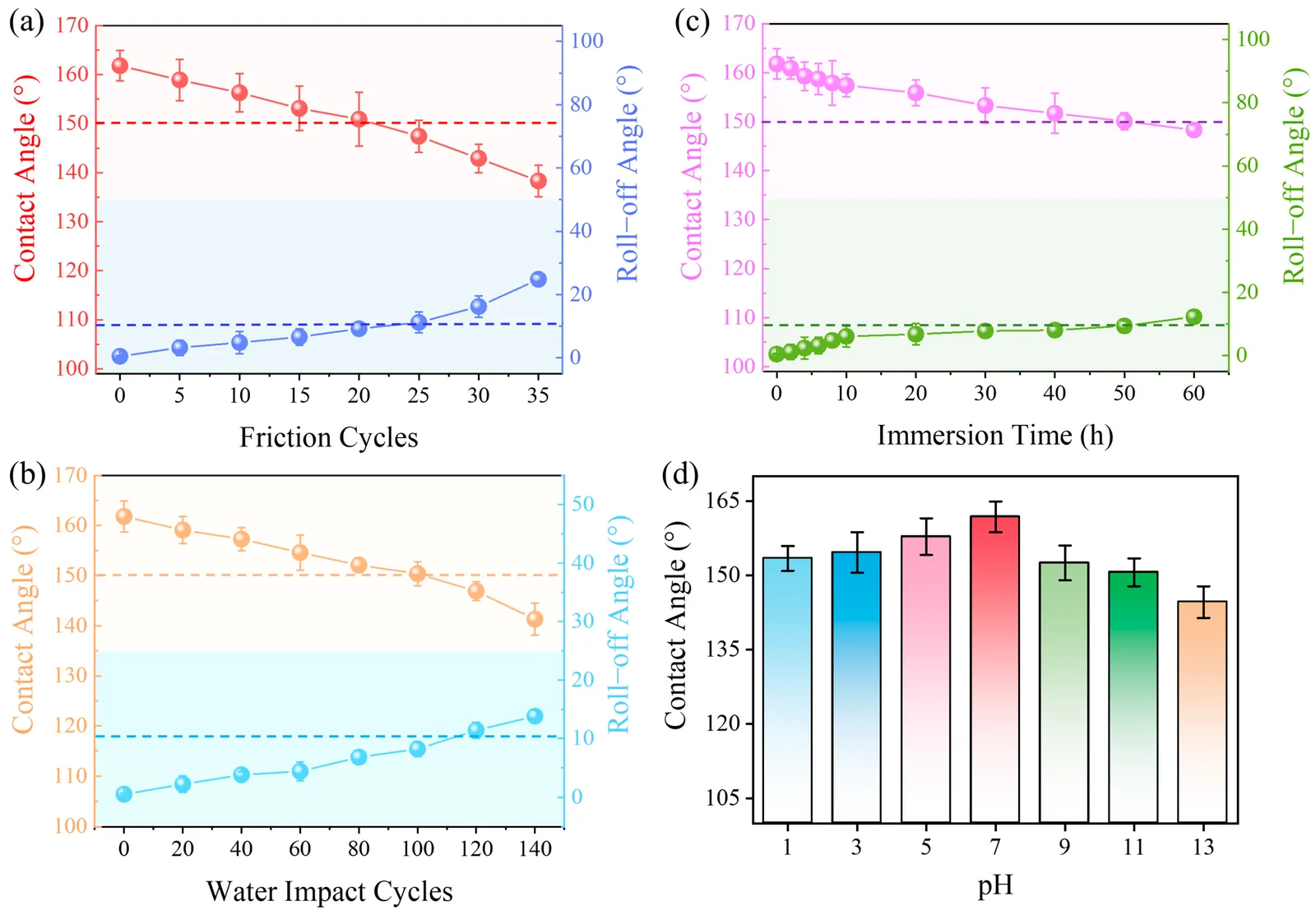
Durability and stability of the superhydrophobic coating: variations of water CA and RA. (**a**) with the number of sandpaper abrasion cycles (1000-grit sandpaper, 200 g load over a 20 mm × 20 mm contact area, 10 cm per cycle); (**b**) with the number of repeated water-entry cycles (a 20 mm coated steel ball released from 10 cm above the water surface); (**c**) with immersion time in DI; and (**d**) with the pH of the immersing solution after 30 min immersion (pH adjusted with 0.1 M HCl and 0.1 M NaOH).

For practical marine applications, a superhydrophobic coating must withstand mechanical wear, prolonged immersion, and corrosive environments without losing its water repellency. The durability of the ZnO/epoxy coating was therefore evaluated through sandpaper abrasion, underwater immersion, repeated water-entry, and chemical stability tests (Figure 2).

As shown in Figure 2a, the coating retained its superhydrophobicity (CA > 150°) during the first 20 abrasion cycles (200 cm), with the CA decreasing slightly from 161.8 ± 3.1° to 150.9 ± 5.5° and the RA increasing from below 1° to 9.2 ± 2.1°. Further abrasion gradually damaged the hierarchical micro/nanostructures: after 35 cycles (350 cm), the CA decreased to 138.3 ± 3.2° and the RA rose to 24.8 ± 1.7°, indicating the loss of superhydrophobicity. This abrasion resistance compares favorably with previously reported superhydrophobic paints that retained their water repellency after 100 cm of sandpaper abrasion [20]. The underwater stability of the coating is presented in Figure 2b. The CA remained above 150° after 50 h of immersion in deionized water (DI) and decreased only slightly to 148.3 ± 1.1° after 60 h, while the RA stayed below 13° throughout the test. This indicates that the air layer trapped within the hierarchical structures is preserved far longer than the thin plastrons that were reported to dissolve within a few hours [23], which is essential for sustained underwater drag reduction. Repeated water-entry tests were conducted to mimic the operating conditions of the underwater free-fall experiments (Figure 2c). The coated sphere remained superhydrophobic after 100 water-entry cycles (CA of 150.4 ± 2.4°), and the CA decreased to 141.3 ± 3.2° after 140 cycles, demonstrating that the coating endures the repeated impact and wetting associated with underwater deployment. Finally, the coating exhibited excellent chemical stability over the full pH range (Figure 2d): the CA exceeded 150° in solutions with pH values from 1 to 11 and remained as high as 144.6 ± 3.2° even at pH 13, which is consistent with the chemical robustness reported for ZnO/epoxy-based coatings [33,34]. The moderate decrease of the contact angle at pH 13 can be rationalized by the amphoteric nature of ZnO, which gradually dissolves in strongly alkaline media as zincate ions (ZnO + 2OH^−^ + H_2_O → [Zn(OH)_4_]^2−^), together with the partial saponification and desorption of the grafted stearic acid under strong alkalinity. Both processes slowly erode the hierarchical texture and the low-surface-energy layer. Nevertheless, the retention of a high contact angle (144.6 ± 3.2°) after 30 min indicates that the epoxy matrix preserves the integrity of the coating during short-term exposure to strongly alkaline media. Together, these results demonstrate a satisfactory laboratory-scale durability of the ZnO/epoxy superhydrophobic coating, which is comparable to the current state of the art of epoxy-based systems. Under abrasive wear, the present coating retained its superhydrophobicity after 200 cm of 1000-grit sandpaper abrasion under a contact pressure of 4.9 kPa; for comparison, the EP/PF@SiO_2_ composite coating reported by Wang et al. [35] withstood a longer abrasion distance (7 m, WCA = 154°) but under a lower pressure (2.45 kPa) and a finer abrasive (2000-grit), and the laser-textured epoxy enamel of Kuzina et al. [36] maintained CA above 150° after 60 min of oscillating-sand abrasion (ASTM F735) [37]. Regarding chemical robustness, the present coating remained superhydrophobic from pH 1 to 11 and retained a CA of 144.6° at pH 13, whereas the above epoxy coatings were validated by long-term corrosion tests (35-day salt spray for the EP/PF@SiO_2_ coating; 100-day continuous contact with 0.5 M NaCl for the laser-textured enamel) that primarily address barrier performance rather than retention of the Cassie state. It should be emphasized, however, that these tests demonstrate laboratory-scale durability rather than readiness for practical marine deployment; long-term validation under sustained hydrodynamic shear, biofouling and realistic seawater chemistry remains necessary, as discussed in the Discussion section.

### 3.3. Drag-Reduction Performance on the Water Surface

**Figure 3:**
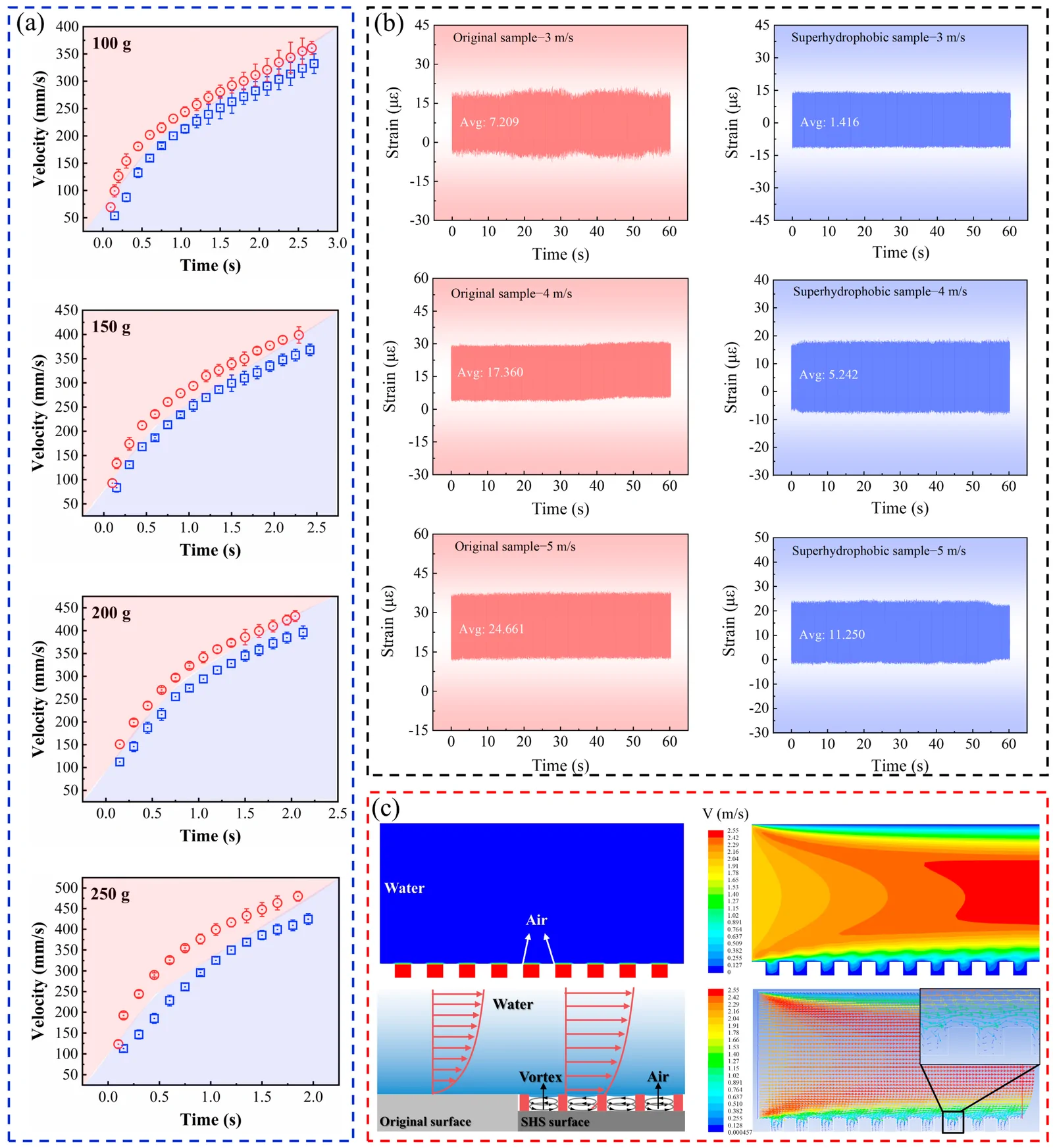
Drag-reduction performance of the superhydrophobic coating on the water surface. (**a**) Velocity–time profiles of the uncoated and superhydrophobic model ships under towing weights of 100–250 g; (**b**) mean strain signals measured on the uncoated and superhydrophobic samples at water flow velocities of 3, 4 and 5 m/s in the solid–liquid interfacial friction test; (**c**) simulated velocity fields of water flowing over smooth (upper wall) and superhydrophobic (lower wall) surfaces.

It is well established that a superhydrophobic surface spontaneously retains an air layer upon immersion, converting the solid–liquid contact into a solid–gas–liquid one and thereby reducing the surface drag [12,13]. The drag-reduction capability of the ZnO/epoxy superhydrophobic coating for surface vehicles was first evaluated by model ship towing tests. The velocity–time profiles of the uncoated and superhydrophobic model ships are compared in Figure 3a. Since the terminal velocity was not fully attained within the 900 mm tank, it was estimated from the measured velocity–time curves as follows. Each curve was smoothed with a fourth-order polynomial, which serves only as a local fit of the recorded data, and the terminal velocity was taken at the point where the slope of the fitted curve vanishes (d*v*/d*t* = 0), i.e., where the acceleration vanishes, which defines the terminal state (Figures S3–S6). For all eight conditions, this zero-slope point lies within or immediately after the recorded window (≤0.4 s beyond the last frame), and the final measured velocities already attain 92–100% of the estimated terminal velocities. Moreover, evaluating Equation (6) directly at the final measured velocities, without any fitting, still yields drag-reduction efficiencies of 15–22%, confirming that the result does not hinge on the fitting procedure. The uncoated ship exhibited terminal velocities of 343.75, 366.73, 407.36 and 446.86 mm/s under towing weights of 100, 150, 200 and 250 g, respectively, whereas the superhydrophobic ship reached higher terminal velocities of 360.42, 397.85, 458.78 and 520.76 mm/s under the same conditions. The corresponding Reynolds numbers, *Re* = *ρU*_T_*L*/*μ* with the characteristic length *L* = 0.15 m, ranged from 51,563 to 67,029 for the uncoated ship and from 54,063 to 78,114 for the superhydrophobic one. In this regime (Re ~ 10^4^–10^5^), frictional resistance constitutes a substantial fraction of the total drag of a small surface vessel. The drag coefficients and drag-reduction efficiencies were then determined from Equations (5) and (6), giving drag-reduction efficiencies of 9%, 15%, 21% and 26%, respectively, showing a clear improvement with increasing towing force and Reynolds number. This enhancement is attributed to the air layer sustained on the superhydrophobic hull, which changes the interfacial condition from solid–liquid to solid–gas–liquid, induces wall slip and reduces the frictional drag, thereby increasing the terminal velocity of the model ship. To compare the two ships at matched velocities, resistance curves were constructed from the four measured (*F*_T_, *U*_T_) pairs for each ship, noting that the resistance at terminal velocity equals the towing force (Figure S7). Power-law fits yield *R* = 37.9*U*^3.34^ for the uncoated ship and *R* = 12.3*U*^2.41^ for the superhydrophobic ship. At matched velocities within the common range (0.360~0.447 m/s), the drag-reduction efficiency ranges from approximately 15% to 31% and increases with velocity, consistent with the trend obtained from the matched-towing-force comparison (9~26% for towing weights of 100~250 g). It should be noted that the resistance-curve comparison is constructed from the same terminal-velocity estimates and is therefore not an independent validation; it provides a matched-velocity view of the same data, while the independent support comes from the raw velocity–time profiles, the model-free evaluation at the final measured velocities, and the interfacial friction and simulation results.

To further quantify the frictional drag reduction at the solid–liquid interface, the weak interfacial frictional forces were measured using the custom-built apparatus, complemented by numerical simulation. The mean strain signals acquired on the uncoated and superhydrophobic samples at flow velocities of 3, 4 and 5 m/s are compared in Figure 3b. The strain of the superhydrophobic sample was markedly lower than that of the uncoated one at all velocities: the measured strains decreased from 7.209, 17.360 and 24.661 με for the uncoated sample to 1.416, 5.242 and 11.250 με for the superhydrophobic one at 3, 4 and 5 m/s, respectively. Substituting these strains into Equations (7)–(12) yields the corresponding frictional forces; at 5 m/s, for example, the frictional force decreased from 0.316 N for the uncoated sample to 0.144 N for the superhydrophobic one. The drag-reduction efficiencies calculated from the strain ratios were 80%, 70% and 54% at 3, 4 and 5 m/s, respectively. The decreasing drag-reduction efficiency with increasing flow velocity is ascribed to the gradual destabilization and partial loss of the trapped air layer under higher wall shear, which weakens the slip effect. These results confirm the remarkable frictional drag-reduction capability of the ZnO/epoxy superhydrophobic coating.

Numerical simulations of water flowing over smooth and superhydrophobic surfaces were performed with FLUENT to verify the above mechanism, with the results summarized in Figure 3c. The computational domain consisted of a smooth flat plate as the upper wall and a superhydrophobic surface model as the lower wall. A simplified bidirectional microgroove structure consistent with the Cassie wetting state was adopted to represent the micro/nano features of the superhydrophobic coating, and local mesh refinement was applied near the walls. The simulated flow field exhibited a clear velocity gradient across the channel. At equivalent distances from the two walls, the flow velocity over the superhydrophobic surface was higher than that over the smooth surface, which is attributed to the air layer trapped within the microstructures inducing interfacial slip. This slip behavior can be described by the Navier boundary condition, in which the slip velocity us is proportional to the local velocity gradient at the wall [38]:

(13) $$u_{0}\;=\;b\left|\frac{\partial u}{\partial y}\right|$$

where *b* is the slip length. In addition, the air layer displaces vortices away from the wall and stabilizes them in the outer flow region, which suppresses vortex–wall interactions and mitigates the near-wall shear stress. Through these combined effects, the superhydrophobic surface significantly attenuates the frictional drag. Quantitatively, the simulated drag forces were 0.061 and 0.036 N for the smooth and superhydrophobic surfaces, respectively, corresponding to a drag-reduction efficiency of 41%, which is consistent with the experimental interfacial friction results and thereby validates the reliability of the measurements.

### 3.4. Underwater Drag-Reduction Performance

**Figure 4:**
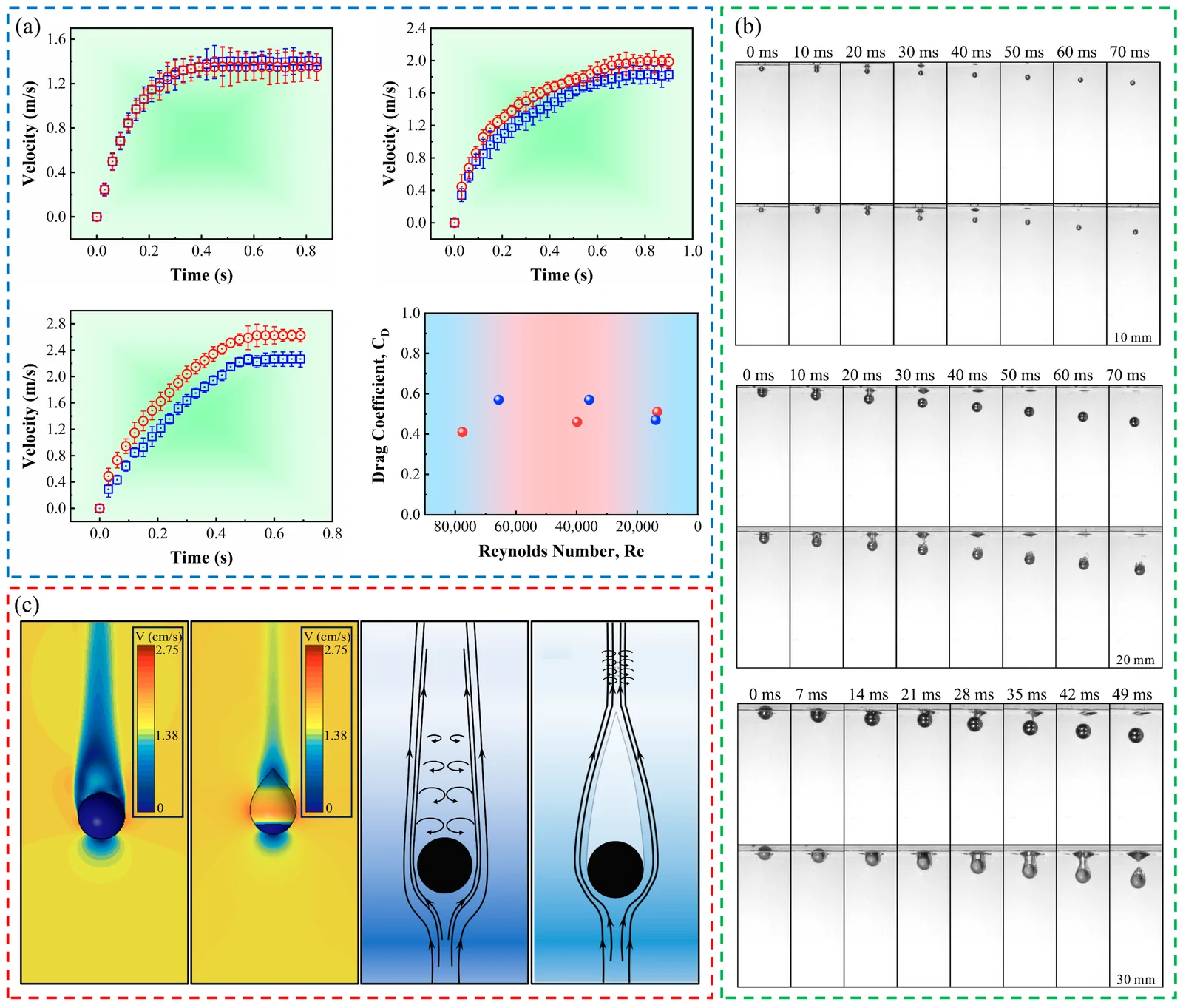
Underwater drag-reduction performance of the superhydrophobic coating. (**a**) Velocity–time profiles of smooth and superhydrophobic steel balls with diameters of 10, 20 and 30 mm during free fall in water; (**b**) high-speed camera snapshots of the water entry of smooth and superhydrophobic balls with diameters of 10, 20 and 30 mm; (**c**) simulated velocity fields around the smooth and cavity-forming 30 mm balls at *Re* = 24,000.

The underwater drag-reduction performance of the coating was evaluated by free-fall tests of steel balls with diameters of 10, 20 and 30 mm. The velocity–time profiles of the smooth and superhydrophobic balls are compared in Figure 4a, showing a pronounced size-dependent behavior. For the 10 mm balls, the smooth specimen accelerated faster and reached a higher terminal velocity (1.40 m/s) than the superhydrophobic one (1.35 m/s). In contrast, for the 20 and 30 mm balls, the superhydrophobic specimens accelerated more rapidly and attained markedly higher terminal velocities (1.99 and 2.63 m/s, respectively) than the smooth ones (1.83 and 2.26 m/s, respectively).

The drag coefficients were calculated from the terminal velocities using Equation (2). For the 10 mm balls, the drag coefficient of the superhydrophobic ball (*C*_D,sh_ = 0.51) was higher than that of the smooth one (*C*_D,s_ = 0.47), with Reynolds numbers of 13,433 and 13,930, respectively, corresponding to a negative drag-reduction efficiency of −9%. At this relatively low Reynolds number (*Re* ≈ 1.4 × 10^4^), the frictional drag accounts for a considerable fraction of the total drag [17], and the added SA of the coating outweighs the beneficial effect of the entrained air layer [36], leading to a net increase in drag. For the 20 and 30 mm balls, the superhydrophobic specimens exhibited lower drag coefficients (*C*_D,sh_ = 0.46 and 0.41) than the smooth ones (*C*_D,s_ = 0.54 for both), with Reynolds numbers ranging from 35,821 to 77,612. In this regime (10^4^ < *Re* < 10^5^), pressure drag becomes dominant [36]. The corresponding drag-reduction efficiencies reached 19% and 28% for the 20 and 30 mm balls, respectively, demonstrating the effectiveness of the coating in reducing both pressure and frictional drag under underwater conditions.

To elucidate the origin of this size-dependent behavior, the interfacial behavior of the balls upon water entry (released at the water surface) was observed by high-speed imaging (Figure 4b and Movies S1–S3). For the 10 mm balls, the smooth specimen entered the water without air entrainment, whereas the superhydrophobic ball captured only a small volume of air: the air–liquid interface deformed slightly and became concave at 30 ms, and the cavity elongated and destabilized during descent, leaving minimal bubbles adhered to the surface (Movie S1). For the 20 mm balls, the smooth specimen again showed no air retention, while the superhydrophobic one trapped a more substantial air layer, causing pronounced interface deformation by 20 ms; the cavity elongated and partially ruptured during descent, but the residual bubbles formed a semi-stable air film on the surface (Movie S2). For the 30 mm balls, the smooth specimen still exhibited no attached bubbles, whereas the superhydrophobic ball entrapped a large volume of air upon impact, producing a marked concavity of the interface by 28 ms; the cavity extended vertically and then contracted under surface tension and hydrostatic pressure into a well-defined spindle shape by 42 ms (Movie S3). The formation of this spindle-shaped cavity is attributed to the larger coated surface area entrapping more air, which stabilizes the Cassie–Baxter state over the entire ball surface.

Combining the drag-reduction data with the cavity observations, the underwater drag-reducing performance of the coating is governed by the morphology and stability of the entrained air cavity: specimens with well-defined spindle-shaped cavities exhibit superior drag reduction compared with those bearing irregular or incomplete air layers. Two mechanisms are responsible. First, the cavity modifies the effective contour of the sphere, altering the surrounding flow field and pressure distribution and thus reducing the pressure drag. Second, the cavity establishes a solid–gas–liquid interface that promotes slip and markedly decreases the frictional drag. To quantitatively verify these mechanisms, the flow around smooth and cavity-forming 30 mm balls was simulated using STAR-CCM+ at a Reynolds number of 24,000, matching the experimental conditions (Figure 4c). For the smooth sphere, the boundary layer separated near the equator owing to the fluid viscosity and the adverse pressure gradient, which was intensified by the high curvature of the aft section; this produced a large recirculation zone with significantly reduced velocity behind the sphere. In contrast, the cavity reduced the effective curvature of the rear surface and mitigated the adverse pressure gradient, so that the flow separation was delayed and the wake region became much smaller, lowering both the frictional and pressure drag. In other words, the cavity acted as a hydrodynamic “shaping agent” that streamlined the sphere, and its combined effects significantly diminished the total hydrodynamic resistance, thereby increasing the descent velocity and validating the efficacy of the superhydrophobic coating for underwater drag reduction.

## 4. Discussion

The present results confirm the working hypothesis that a single superhydrophobic coating can reduce hydrodynamic drag in both navigation modes, albeit through two distinct mechanisms governed by the same air-retention principle. On the water surface, the trapped air layer converts the solid–liquid contact into a solid–gas–liquid one and induces wall slip, as evidenced directly by the reduced interfacial frictional force (Figure 3b) and by the higher near-wall velocity in the FLUENT simulations (Figure 3c). This interpretation is consistent with the slip-length picture established for superhydrophobic surfaces in laminar channel flows [12,14,15] and with previous model-ship studies reporting drag-reduction efficiencies of 12.7–16.8% [19,20,21]. The higher efficiencies obtained here (up to 31% at matched velocities) may be associated with the hierarchical flower-like micro/nanostructure, which stabilizes a continuous air layer over the entire wetted hull, and with the low-Reynolds-number regime in which frictional drag dominates. It should nevertheless be noted that the absolute drag of small model ships is sensitive to wetted-area and trim conditions, and the present values should be regarded as an upper bound pending validation at larger scales.

Underwater, the drag reduction exhibits a pronounced size dependence that can be rationalized by the competition between pressure drag and frictional drag across Reynolds regimes. For the 10 mm ball (*Re* ≈ 1.4 × 10^4^), the coating increased the drag by 9%, because the added roughness raises the frictional component while the small entrained air volume cannot form a stable cavity—consistent with the observations of Jetly et al. [23] that the benefit of a thin air layer on small spheres is marginal unless a cavity or a drag-crisis condition is triggered [11]. For the 20 and 30 mm balls (Re of 10^4^–10^5^), the entrained spindle-shaped cavity acted as a hydrodynamic “shaping agent”, delaying flow separation and shrinking the wake, thereby reducing the dominant pressure drag. This mechanism complements the drag-reduction route demonstrated for superhydrophobic submarine models, in which an enveloping air film mainly reduces skin friction [22], and suggests that cavity-assisted pressure-drag reduction may be particularly effective for blunt underwater bodies of intermediate size. An important practical implication is that the coating does not need to remain perfectly superhydrophobic over the entire surface: even a partially ruptured cavity, as observed for the 20 mm ball, still yielded a 19% drag reduction.

The durability results (Figure 2) address the most frequently raised obstacle to the practical deployment of superhydrophobic coatings [31]. The coating retained its superhydrophobicity after 200 cm of sandpaper abrasion, 50 h of underwater immersion, 100 repeated water-entry cycles, and exposure to pH 1–11 solutions. This robustness is attributed to the combination of the mechanically interlocked ZnO/epoxy matrix and the re-exposure of low-surface-energy stearic acid moieties upon mild wear, in line with the behavior reported for chemically stable ZnO/epoxy-based coatings [33,34]. Nevertheless, the present durability tests were conducted under laboratory conditions and over limited durations; the long-term stability of the air layer under sustained hydrodynamic shear, biofouling, and realistic seawater chemistry remains to be verified. Furthermore, all experiments were performed at laboratory scales and moderate Reynolds numbers, and the extrapolation of the present drag-reduction efficiencies to full-scale vessels operating in turbulent regimes requires dedicated studies, given the known sensitivity of air-layer stability to pressure and flow velocity.

Future work will therefore focus on (i) long-term field tests of coated hulls and underwater bodies in realistic seawater environments; (ii) further improvement of the mechanical robustness of the coating, for example through self-healing chemistries or hierarchical reinforcement; and (iii) coupling the coating with complementary drag-reduction strategies, such as microbubble injection [27] or biomimetic riblet textures [28], toward integrated energy-saving solutions for marine vehicles.

## 5. Materials and Methods

### 5.1. Materials

Model boats (135 × 85 × 55 mm^3^) and steel balls with diameters of 10, 20 and 30 mm were used as substrates. Epoxy resin (E51) and its curing agent (593) were supplied by Shanghai Aotun Chemical Technology Co., Ltd., Shanghai, China. ZnO powder (particle size of 2–20 μm, purity ≥ 98%), stearic acid, acetic acid, ethanol and acetone were purchased from Chengdu Kelong Chemical Co., Ltd., Chengdu, China. All reagents were of analytical grade and used as received. DI was used throughout all experiments.

### 5.2. Preparation of Superhydrophobic Coatings

The superhydrophobic coating was fabricated following our previously reported procedure [34]. Substrates (model boats, steel balls and flat plates) were ultrasonically cleaned in acetone and ethanol for 15 min to remove surface contaminants, and then dried in air. A precursor suspension was prepared by mixing 8 g of ZnO powder with epoxy resin, curing agent and ethanol at a mass ratio of 3:1:9, followed by magnetic stirring for 30 min at room temperature. The homogeneous suspension was spray-coated onto the substrates using an air spray gun operated at an air pressure of 0.25 MPa and a nozzle-to-substrate distance of 25 cm. The coated samples were then cured in an oven at 75 °C for 6 h to obtain the original ZnO/epoxy coating. Finally, the samples were immersed in a mixed solution of stearic acid, acetic acid and ethanol for 8–30 min to reduce the surface energy, rinsed with ethanol and dried in air, yielding the superhydrophobic ZnO/epoxy coating. Unless otherwise stated, an immersion time of 18 min was adopted, at which the coating exhibited the highest water CA (see Section 3.1).

### 5.3. Characterization

The surface morphology and elemental composition of the coatings were examined by SEM (JSM-7800F, JEOL, Tokyo, Japan) equipped with an EDS. The chemical functional groups were identified by FTIR (Nicolet iS50, Thermo Fisher Scientific, Waltham, MA, USA), and the surface chemical states were analyzed byXPS. The water CA and RA were measured using a contact-angle goniometer (SDC-350H, Sindin Precision Instrument Co., Ltd., Dongguan, China) with 5.0 μL water droplets at room temperature. Each reported CA/RA value is the average of at least five measurements taken at different positions on each sample. The surface roughness was measured by white-light interferometry (Contour GT-X 3D Optical Microscope, Bruker Nano Surface, Karlsruhe, Germany) over an area of 4.56 × 3.42 mm^2^, the areal height parameters (Sa, Rq, Rt) were evaluated according to ISO 25178 [39].

### 5.4. Drag Reduction Test

#### 5.4.1. Model Ship Towing Test

**Figure 5:**
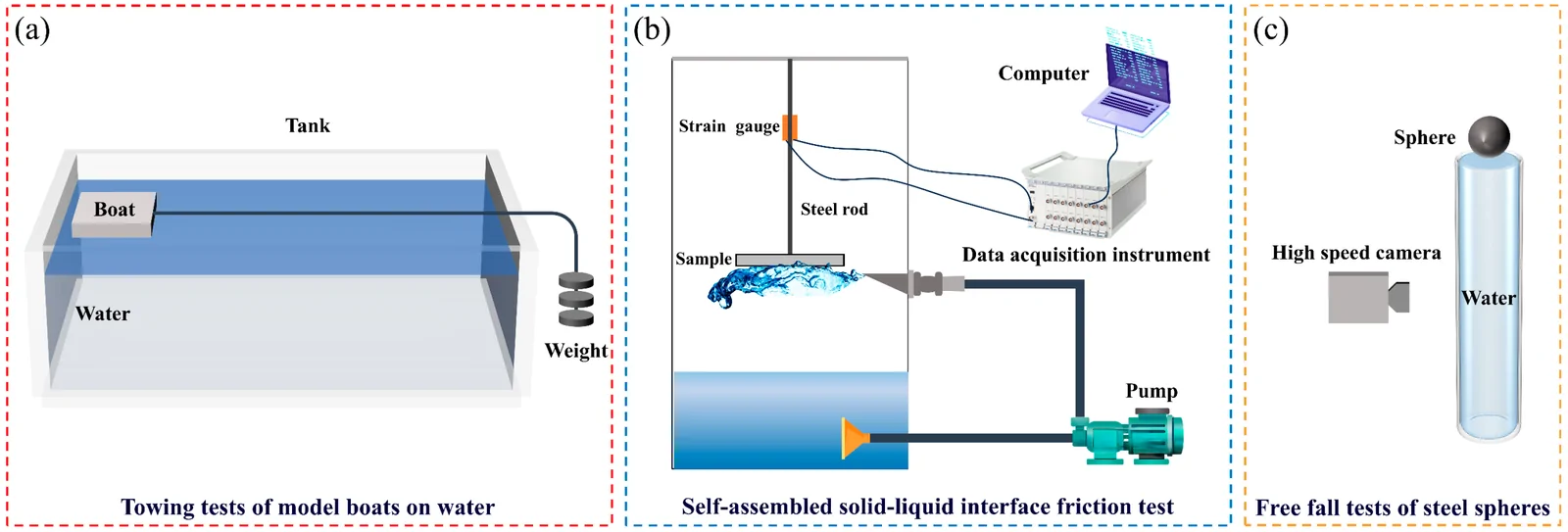
Experimental setups for the drag-reduction tests. (**a**) Model ship towing test on the water surface; (**b**) custom-built solid–liquid interfacial friction test; (**c**) underwater free-fall test of steel balls.

The drag-reduction performance of the coating for surface vehicles was evaluated by a model ship towing test (Figure 5a). A 70 g iron plate was placed inside the model ship as ballast to maintain a constant draft. The ship was connected via a rope and a pulley to a hanging weight (100, 150, 200 or 250 g), whose descent drove the ship along a 900 mm-long water tank. The motion was recorded by a digital camera, and the trajectory was extracted frame by frame to obtain the instantaneous and terminal velocities. Each terminal velocity value represents the average of at least three independent runs.

#### 5.4.2. Solid–Liquid Interfacial Friction Test

A custom-built apparatus was used to measure the weak frictional force at the solid–liquid interface (Figure 5b). The apparatus consists of a water tank, a water pump, a flow meter, a nozzle, a cantilever rod, a strain gauge (BF120-3AA-P500-D, ZEMIC, Hanzhong, China; resistance 119.8 ± 0.3 Ω; gauge factor 2.0 ± 1%) and a dynamic signal acquisition system (DH5922N, Donghua Testing Technology Co., Ltd., Jingjiang, China). During the test, water was pumped from the tank and ejected from the nozzle tangentially along the sample surface (wall-jet configuration, with the nozzle axis parallel to the sample) at controlled velocities of 3, 4 and 5 m/s; the strain gauge records the bending of the cantilever, i.e., the tangential (shear) force acting on the sample. The resulting frictional force deflected the sample and deformed the strain gauge, whose output signal was recorded by the acquisition system and converted into the frictional force according to Equations (7)–(12).

#### 5.4.3. Underwater Free-Fall Test

The underwater drag-reduction performance was evaluated by free-fall tests of steel balls (Figure 5c). A transparent cylindrical tank (diameter 500 mm × height 1200 mm) was filled with DI at room temperature. Steel balls with diameters of 10, 20 and 30 mm, with and without the superhydrophobic coating, were released from rest at the water surface. The descending motion was recorded simultaneously by a digital camera and a high-speed camera. The recorded videos were analyzed frame by frame to extract trajectories and to derive instantaneous and terminal velocities, from which the drag coefficient and drag-reduction efficiency were calculated (Section 2.1). Each reported velocity is the mean of no fewer than three independent trials.

### 5.5. Durability and Stability Tests

The mechanical durability of the coating was evaluated through sandpaper abrasion tests following a standard method. The coated flat sample was placed face down on 1000-grit sandpaper under a 200 g weight (contact area of 20 mm × 20 mm) and dragged for 10 cm per cycle; the CA and RA were measured after every five cycles. The underwater stability was assessed by immersing the coated samples in DI for up to 60 h, with the wettability measured at selected time intervals. Repeated water-entry tests were performed by releasing a coated steel sphere (20 mm in diameter) from 10 cm above the water surface; after a given number of cycles, the sphere was retrieved and gently wiped dry before the wettability measurement. The chemical stability was examined by immersing the coated samples in aqueous solutions with pH values ranging from 1 to 13 (adjusted using 0.1 M HCl and 0.1 M NaOH solutions) for 30 min; the samples were then wiped dry and subjected to CA measurements.

## 6. Conclusions

A durable ZnO/epoxy superhydrophobic coating (CA = 161.8 ± 3.1°, RA < 1°) was fabricated by a facile route combining spray coating and chemical immersion, and its dual-mode drag-reduction performance was demonstrated for both surface and underwater vehicles. The coating remained superhydrophobic (CA > 150°) after 200 cm of sandpaper abrasion, 50 h of underwater immersion, 100 repeated water-entry cycles and immersion in pH 1–11 solutions. For surface vehicles, the coating increased the terminal velocity of a model ship under towing weights of 100~250 g, achieving drag-reduction efficiencies of 9~26% under matched towing forces and up to 31% at matched velocities; interfacial friction measurements and FLUENT simulations consistently attributed this enhancement to the slip induced by the retained air layer. For underwater vehicles, the drag reduction was governed by the entrained air cavity: the 20 and 30 mm superhydrophobic balls achieved drag-reduction efficiencies of 19% and 28%, respectively, whereas the 10 mm ball showed a 9% drag increase due to the dominant roughness effect at low Reynolds number. High-speed imaging and STAR-CCM+ simulations revealed that the stable spindle-shaped cavity delays flow separation and acts as a hydrodynamic “shaping agent”, reducing both pressure and frictional drag. These results provide a feasible coating strategy for energy-saving marine vehicles operating at or below the water surface.

## Data Availability

Data will be made available on request.

## Supplementary Materials

The following supporting information can be downloaded at https://www.mdpi.com/article/10.3390/molecules31213896/s1, Figure S1: White-light interferometry results of the superhydrophobic coating (immersion time of 18 min): (a) 3D surface topography, (b) 2D height map, and (c) height distribution histogram with Gaussian fit, measured over an area of 4.56 × 3.42 mm^2^ (Sa = 12.1 μm, Rq = 15.1 μm, Rt = 107.6 μm); Figure S2: High-magnification SEM image of the superhydrophobic coating, showing the interconnected porous nanosheet network (wall thicknesses on the order of tens of nanometers) assembling the hierarchical microstructure; Figure S3: Fitted velocity–time curves of the (a) superhydrophobic and (b) uncoated model boats under a towing weight of 100 g; Figure S4: Fitted velocity–time curves of the (a) superhydrophobic and (b) uncoated model boats under a towing weight of 150 g; Figure S5: Fitted velocity–time curves of the (a) superhydrophobic and (b) uncoated model boats under a towing weight of 200 g; Figure S6: Fitted velocity–time curves of the (a) superhydrophobic and (b) uncoated model boats under a towing weight of 250 g; Figure S7: Resistance curves of the uncoated and superhydrophobic model ships constructed from the measured data points, with power-law fits. The shaded region indicates the common velocity range used for the matched-velocity comparison; Movie S1: Free-fall process of the 10 mm smooth and superhydrophobic steel balls in water; Movie S2: Free-fall process of the 20 mm smooth and superhydrophobic steel balls in water; Movie S3: Free-fall process of the 30 mm smooth and superhydrophobic steel balls in water.

## Abbreviations

The following abbreviations are used in this manuscript:

CA	Water contact angle
C_D_	Drag coefficient
DI	Deionized water
DR	Drag-reduction efficiency
EDS	Energy-dispersive X-ray spectroscopy
SEM	Field-emission scanning electron microscopy
FTIR	Fourier-transform infrared spectroscopy
Re	Reynolds number
RA	Roll-off angle
Sa	Surface roughness
XPS	X-ray photoelectron spectroscopy
