# Supplementary material for: A Durable Hierarchical ZnO/Epoxy Superhydrophobic Coating for Dual-Mode Drag Reduction of Surface and Underwater Vehicles
Source: Molecules. 2026 Oct 28;31(21):3896. doi: 10.3390/molecules31213896 (PMC13765053; doi:10.3390/molecules31213896)
Supplement: Supplementary file 1 [file molecules-31-03896-s001.zip › Supplementary Material.pdf]

Supplementary Material

A durable hierarchical ZnO/epoxy superhydrophobic coating for dual-mode drag reduction of surface and underwater vehicles

Ruoyu Sun[1], Yan Huang[2], Jing Zhao[3], Jiangpeng Qu[3]*

[1] School of Mechanical Engineering, Sichuan University of Science and Engineering, Yibin, Sichuan, China

[2] College of Physical Education and Health Science, Yibin University, Yibin, Sichuan, China

[3]School of Mechanical Engineering, Southwest Jiaotong University, Chengdu, Sichuan, China

** Corresponding author. E-mail address: sunry@suse.edu.cn (Ruoyu Sun)*

Supplementary movie

Supplementary movie S1. Free-fall process of the 10 mm smooth and superhydrophobic steel balls in water.

Supplementary movie S2. Free-fall process of the 20 mm smooth and superhydrophobic steel balls in water.

Supplementary movie S3. Free-fall process of the 30 mm smooth and superhydrophobic steel balls in water.

Supplementary figure

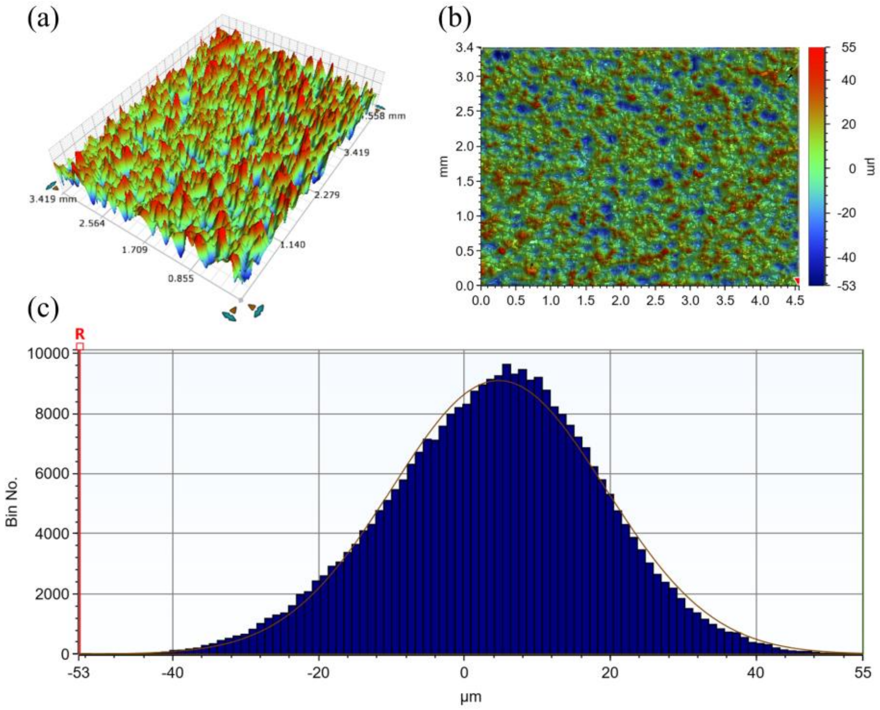

Fig. S1. White-light interferometry results of the superhydrophobic coating (immersion time of 18 min): (a) 3D surface topography, (b) 2D height map, and (c) height distribution histogram with Gaussian fit, measured over an area of 4.56 × 3.42 mm^2 (Sa = 12.1 μm, Rq = 15.1 μm, Rt = 107.6 μm).

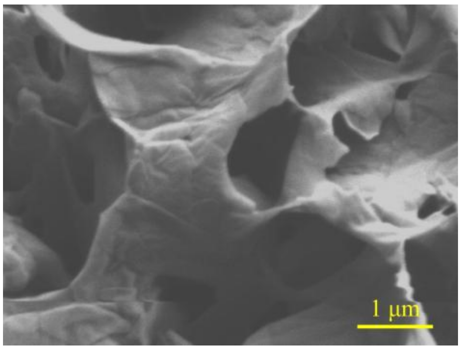

Fig. S2. High-magnification SEM image of the superhydrophobic coating, showing the interconnected porous nanosheet network (wall thicknesses on the order of tens of nanometers) assembling the hierarchical microstructure.

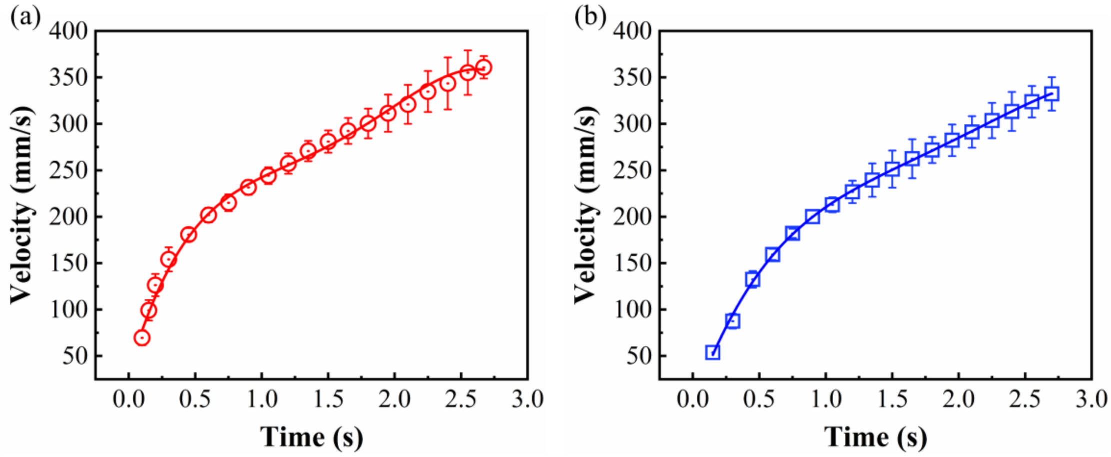

Fig. S3. Fitted velocity–time curves of the (a) superhydrophobic and (b) uncoated model boats under a towing weight of 100 g

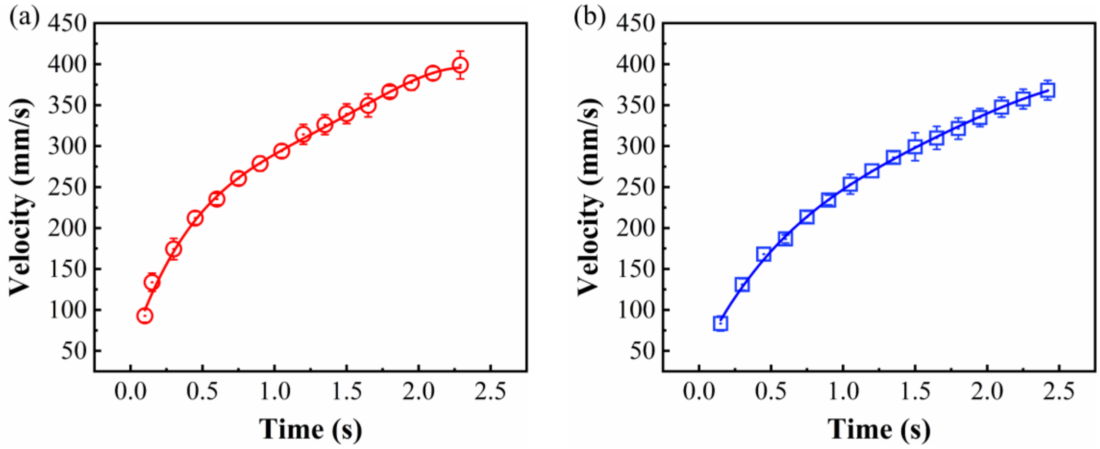

Fig. S4. Fitted velocity–time curves of the (a) superhydrophobic and (b) uncoated model boats under a towing weight of 150 g

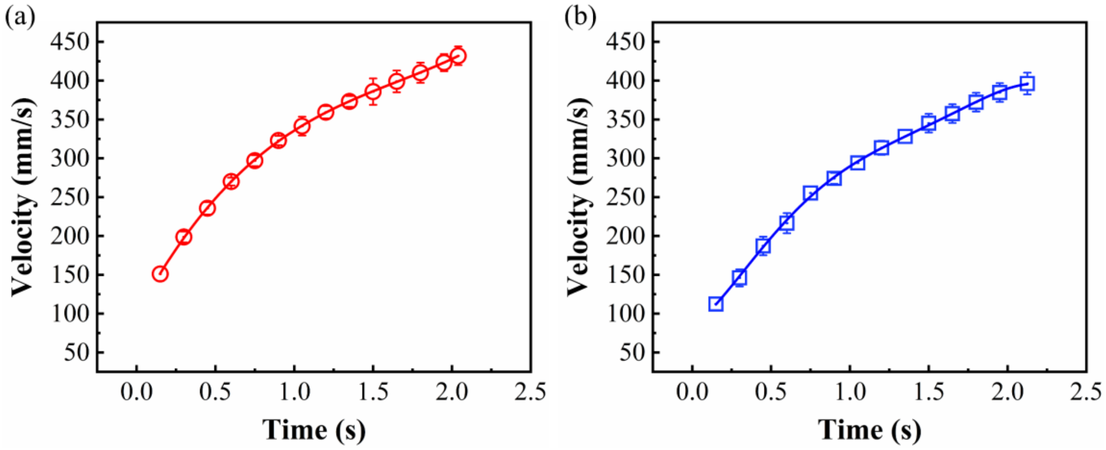

Fig. S5. Fitted velocity–time curves of the (a) superhydrophobic and (b) uncoated model boats under a towing weight of 200 g

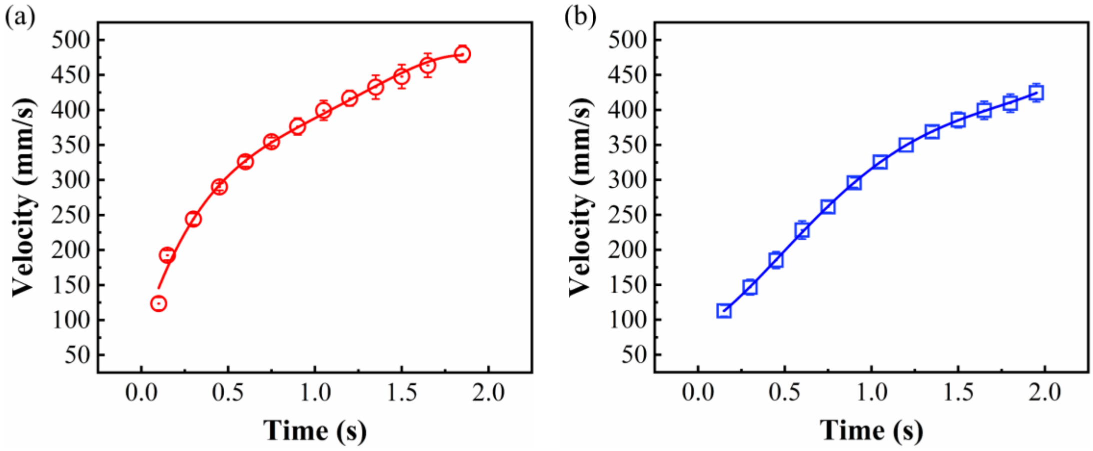

Fig. S6. Fitted velocity–time curves of the (a) superhydrophobic and (b) uncoated model boats under a towing weight of 250 g

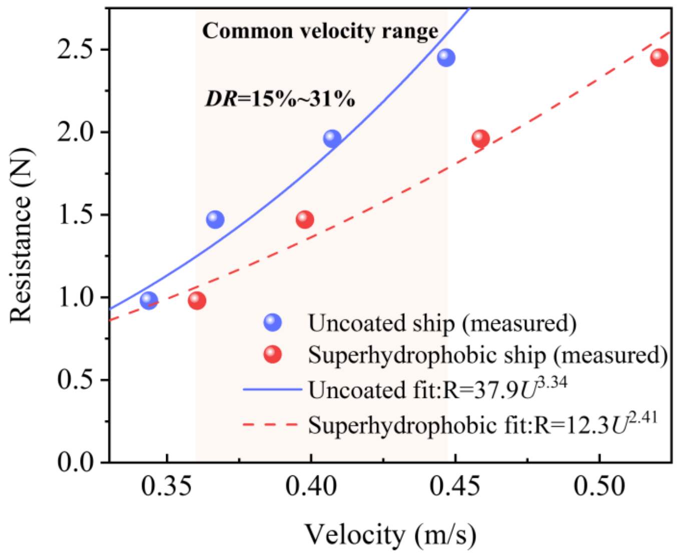

Fig. S7. Resistance curves of the uncoated and superhydrophobic model ships constructed from the measured data points, with power-law fits. The shaded region indicates the common velocity range used for the matched-velocity comparison.
